# Bioactive Resin Cement Color Stability and Restoration Thickness as Determinants of the Final Shade in a Glass–Ceramic CAD/CAM Material

**DOI:** 10.3390/jfb16090319

**Published:** 2025-08-31

**Authors:** Hanin E. Yeslam, Alaa Turkistani

**Affiliations:** 1 Department of Restorative Dentistry, Faculty of Dentistry, King Abdulaziz University, Jeddah 21589, Saudi Arabia; 2 Advanced Technology Dental Research Laboratory, King Abdulaziz University, Jeddah 21589, Saudi Arabia

**Keywords:** bioactive, glass–ceramic, CAD/CAM, resin cements, color, whiteness

## Abstract

Bioactive resin cements are gaining popularity for their clinical benefits, but concerns remain regarding their color stability. This study evaluated the color change (ΔE_00_) and whiteness change (ΔWI_D_) in bioactive resin cements and how their potential discoloration affects the shade of bonded CAD/CAM glass–ceramics at different ceramic thicknesses. VITA Mark II blocks were prepared in three thicknesses (0.5, 0.8, and 1.0 mm) and divided by resin cement: Panavia SA Universal (Pn), Predicta Bioactive Cement (Pr), and ACTIVA BioACTIVE Cement (Ac) (n = 10). Additionally, 10 specimens (10 × 2 mm) of each cement alone were prepared. Color was measured before and after 24 days of coffee immersion. Cement type significantly affected ΔE_00_ (*p* < 0.001). Pn had the highest color stability, followed by Pr and Ac, with significant differences between each. ΔWI_D_ also varied by cement (*p* = 0.004), with Pn and Pr differing significantly from Ac. Ceramic thickness alone had no significant effect on ΔE_00_ or ΔWI_D_, but its interaction with cement type was significant (*p* < 0.001). While ceramic thickness does not directly affect the final shade, its combination with resin cement does. Choosing the right resin cement is key for long-term esthetic outcomes.

## 1. Introduction

Resin cements (RCs) play an essential role in the success of adhesive dentistry by securely bonding esthetic restorations [1]. Recent advancements in their formulations enhance adhesion and long-term dental health and reduce sensitivity, thereby facilitating successful treatment results [2]. Bioactive RCs are increasingly used for cementing esthetic indirect dental restorations, such as veneers, crowns, and fixed partial dentures (FPDs) [3,4]. The term “bioactive” has gained much attention in recent years and is strategically used by manufacturers to promote various dental restorative materials [5]. This has raised controversy in the literature regarding the proper use of the term as it relates to dentistry [6]. The latest FDI policy statement defined bioactive dental materials as materials that have bioactive properties (biological, chemical, or mixed mechanism of action) without diverse effects on body tissues or the prime intended use of the material, which is the restoration of the form and function of missing tooth structure [7].

Recently introduced dental bioactive RCs contain bioactive glasses and calcium silicates that promote chemical bonding and tissue formation, as well as the formation of hydroxyapatite on their surface when in contact with biological fluids [8,9]. Additionally, some bioactive RCs contain compounds with antimicrobial properties such as zinc and magnesium oxide nanoparticles, 2-methacryloyloxyethyl phosphorylcholine (MPC), and other polymeric nanoparticles [10]. Meanwhile, others release fluoride, calcium, and phosphate ions that may also produce an antibacterial effect [8,9,11]. These cements are usually self-adhesive, bonding chemically to the tooth structure and enhancing the durability of the restoration [12,13]. Predicta Bioactive Cement (Parkell, Inc., Edgewood, NY, USA) was recently introduced as a promising bioactive RC that induces the deposition of a thick layer of hydroxyapatite with ion recharge potential [14]. Such bioactive RCs contain methacryloyloxydecyl dihydrogen phosphate (MDP) and bond firmly to ceramic restorations [15]. Another widely used bioactive RC is ACTIVA BioACTIVE cement (Pulpdent Corporation, Watertown, MA, USA), which integrates silica glass particles within a matrix enriched with calcium, phosphate, and fluoride, to enhance its durability, bacterial and microleakage resistance, and bonding to dentin [4,9,16].

All-ceramic, full- and partial-coverage restorations fabricated using computer-aided design/computer-aided manufacturing technology (CAD/CAM) are popular for their convenience, durability, and esthetically pleasing, nature-like appearance [17,18]. Conservative tooth preparations yield thinner, more translucent restorations (0.3–1.0 mm for veneers) that may be influenced by the color of the underlying cement and tooth structure [16,19,20]. Bonded restorations are designed to mimic natural teeth; however, they can be influenced by factors related to cementation and restoration, which impact their appearance, strength, and overall performance [21,22]. RCs bind effectively to the tooth structure, but they may impact the final color of the bonded restoration, which is especially challenging in cases with thin restorations or those with restorations fabricated from highly translucent ceramic materials, where thicker cement layers might drastically affect the overall esthetic outcome [23,24]. The chemical composition of an RC, such as monomers, fillers, opacifiers, and initiators, can influence how light is transmitted through it [1,3] and eventually influence the color of both the cement and bonded restoration [23,25]. The RC’s color can also be affected by intraoral thermal changes, stains, pH fluctuations, and various salivary factors [3,23,26,27,28]. Resin cements may discolor, marginally microleak, and/or eventually fail as a result of resinous hydrolytic changes, water and stain absorption, and material degradation in response to faced challenges [29,30]. Gingival crevicular fluid and various foods and drinks with different temperatures, pigments, and acidity levels further challenge the color stability of RCs and bonded restorations. [4,28,31].

A number of recent studies investigated the color stability of bioactive RCs and restorative materials [3,4,32,33,34,35]. However, most of these studies concentrated on the resin cement material itself. Furthermore, no studies have investigated the effect of the color stability of bioactive RCs—especially the newly introduced Predicta Bioactive Cement—on the final color and whiteness of a CAD/CAM glass–ceramic restoration with different ceramic thicknesses. Considering the influence of bioactive RCs’ shade and color on the esthetic quality of CAD/CAM ceramic restorations, and the show-through effect of discolored cement causing restorative color mismatch, the current study aimed at evaluating the color stability of bioactive RCs. It also aimed at assessing the effect of the potential discoloration of two bioactive and one non-bioactive RC on the final color and whiteness of a bonded glass–ceramic material for CAD/CAM fabrication, comparing three ceramic thicknesses. The null hypotheses were that there was no significant difference in the color stability between the two bioactive (Predicta Bioactive [Pr] and ACTIVA BioACTIVE [Ac]) cements and a non-bioactive (Panavia SA Universal [Pn]) cement; that there was no significant effect of ceramic thickness on the final color and whiteness of the bonded CAD/CAM glass–ceramic material when using either the two bioactive (Pr and Ac) or the non-bioactive (Pn) RC; and that the type of RC (bioactive vs. non-bioactive) did not significantly affect the final color and whiteness of the bonded CAD/CAM glass–ceramic material, irrespective of ceramic thickness.

## 2. Materials and Methods

### 2.1. Study Design

**Figure 1:**
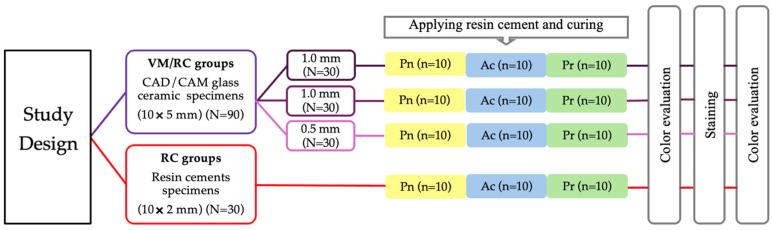
Design of this study. Here, VM/RC is Vita Mark II bonded to resin cement; RC is resin cement; Pn is Panavia resin cement; Ac is Activa BioACTIVE cement; Pr is Predicta Bioactive cement.

A total of 90 blocks specimens of a CAD/CAM feldspar glass–ceramic material (Shade A1 Vita Mark II [VM], Vita Zahnfabrik, Bad Säckingen, Germany) measuring 5 × 5 mm in surface area were prepared for this study. These specimens were randomly divided into three main groups based on ceramic thickness (N = 30): 1.0 mm, 0.8 mm, and 0.5 mm. Each thickness group was further subdivided into three subgroups (n = 10) according to the type of dual-cure resin cement (RC) used. The types included a non-bioactive RC (Panavia SA Universal, Kuraray Noritake Dental, Tokyo, Japan) and two bioactive RCs—Predicta Bioactive Cement (Parkell, Inc., Edgewood, NY, USA) and ACTIVA BioACTIVE Cement (Pulpdent Corporation, Watertown, MA, USA). The color parameters of all bonded specimens were recorded before immersion in a coffee solution for 24 days. After staining, the color parameters were re-recorded to evaluate changes in color and whiteness. Additionally, ten cylindrical specimens (10 × 2 mm) were created from each RC material (total N = 30), and their color parameters were also recorded. These specimens underwent the same immersion process in the coffee solution for 24 days [36], and color changes, along with the whiteness index for each resin material, were assessed. A schematic diagram displaying the study design is shown in Figure 1.

### 2.2. Specimen Preparation for the Evaluation of Ceramic Color with Resin Cement (VM/RC Groups)

A precision low-speed saw cutting machine (TechCut 4TM precision low-speed saw, Allied High-Tech Products, Inc., Rancho Dominguez, CA, USA) was used to cut VM CAD/CAM glass–ceramic blocks using a diamond-coated saw blade (Super-Thin Rim Lapidary saw blade (100 mm in diameter and 0.25 mm in thickness), Jingling, China) under constant water flow. Each milling block was sliced into 1.5 ± 0.1 mm thick plates; then the plates were cut into 5 × 5 × 1.5 ± 0.1 mm rectangular specimens. These were then divided into three groups of 30 each, based on the desired specimen thickness, using the following procedure: Silicon carbide (SiC) paper (300 and 400 μm grits) was used on a rotating polishing machine (EcoMet 30, Buehler, Lake Bluff, IL, USA) to reduce the thickness of the square specimens to approximately 1.1, 0.9, and 0.6 ± 0.1 mm under ample water cooling. Subsequently, finishing and polishing were performed using wet SiC paper of grit sizes 600, 800, 1200, and 2400 μm to eventually achieve specimens with a consistent thickness of 1.0, 0.8, and 0.5 ± 0.1 mm. The polished surface would eliminate surface irregularities that could influence staining susceptibility and color readings [19,37]. The dimensions for all specimens were confirmed using a digital caliper (vernier caliper 200 mm/8 in, Hi-Wendy, New Taipei, Taiwan). All specimens were visually inspected for deformities, fracture lines, and cracks under 2.5× magnification with adequate illumination, and defective ones were discarded.

**Figure 2:**
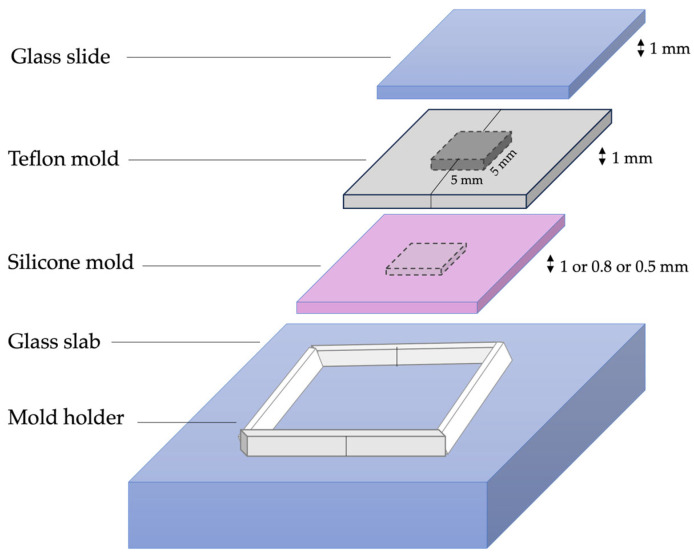
Specimen forming mold for Vita Mark II bonded to resin cement specimens.

The VM ceramic surfaces to be in contact with the RCs were etched with 5% hydrofluoric acid for 60 s, rinsed with distilled water, and then dried using a 3-way syringe. The cementation process was completed using a specially designed Teflon mold with a square hollow measuring 5 × 5 × 1 mm, placed on top of one of three specially designed silicone molds to ensure a uniform 1 mm thick RC layer across all specimens. Each silicone mold contained a 5 × 5 mm hollow with a different depth (1.0, 0.8, or 0.5 mm) depending on the thickness of the ceramic specimen being cemented. The VM/RC specimens’ fabrication molds are shown in Figure 2.

The Teflon/silicone mold and VM specimen combination was placed on a glass slab, and then RC was applied into the Teflon mold’s hollow and covered with a 1 mm thick glass slide, using gentle pressure to squeeze excess cement out and ensure a flat, bubble-free RC surface. Each RC was mixed according to the manufacturer’s instructions, as detailed in Table 1. Curing was performed using a light-emitting diode (LED) curing unit (E-Morlit, Apoza, New Taipei, Taiwan, ROC) for 20 s while maintaining positive pressure on the glass slide for 2 min. A spectroradiometer was used to verify the curing unit’s irradiance, ensuring a consistent level of 1200 mW/cm^2^. The specimen was then carefully removed from the mold, and each side was cured again with overlapping cycles of 20 s to ensure adequate polymerization. Specimens were left undisturbed for 5 min after the light curing cycles, then stored in dark, distilled water containers for 24 h to ensure the full setting of the RCs.

### 2.3. Specimen Preparation for the Evaluation of Cement Color (RC Groups)

Disk-shaped RC specimens (10 × 2 mm) were prepared from three self-adhesive RCs—one conventional dual-cure RC (Pn) and two dual-cure bioactive RCs (AC and PR), with 10 samples each—using a custom Teflon rectangular mold (1 mm thick) with a central circular hollow. The composition and mixing instructions of each investigated RC are detailed in Table 1.

**Table 1:** An overview of the glass–ceramic material and the resin cements investigated in this study.

Material	Ab.	Type	Manufacturer (Lot #)	Composition	Manufacturer’s Instructions
VITA Mark II Blocks	VM	Fine-structure feldspar glass ceramic blocks for CAD/CAM	Vita Zahnfabrik GmbH, Bad Säckingen, Germany	Feldspathic crystal particles in a glass matrix. (SiO_2_. 56–64%. Al_2_O_3_. 20–23%. Na_2_O. 6–9%. K_2_O. 6–8%. CaO. 0.3–0.6%. TiO_2_. 0.0–0.1%.) [38]	Degrease the prepared ceramic restoration using ethanol. Condition the fitting surface with 5% hydrofluoric acid for 60 s, rinse for 60 s, dry for 20 s. Apply silane coupling agent and allow to dry.
Panavia SA Universal (shade A2)	Pn	Non-bioactive, self-adhesive dual-cure resin cement	Kuraray Noritake Dental, Tokyo, Japan (#140200)	Two-paste system, hand mix (62% wt filler loading): Paste A: MDP, Bis-GMA, TEGDMA, hydrophobic aromatic dimethacrylate, HEMA, silanated barium glass filler, silanated colloidal silica, dl-camphorquinone, peroxide, catalysts, pigments. Paste B: hydrophobic aromatic dimethacrylate, silane coupling agent, silanated barium glass filler, aluminum oxide filler, surface-treated sodium fluoride (<1%), dl-camphorquinone, accelerators, pigments	Dispense equal amounts of pastes A and B, mix for 10 s. Apply and tack light cure for 2–5 s, remove excess, and light cure for 10 s.
ACTIVA BioACTIVE Cement (shade A2)	Ac	Bioactive, self-adhesive dual-cure resin cement	Pulpdent, Watertown, MA, USA (#221118)	Dual-paste syringe with auto-mix tips: diurethane and other methacrylates with modified polyacrylic acid (52.9%), silica (5.1%), sodium fluoride (0.9%)	Use auto-mix tip, dispense into restoration, and tack light cure for 1–2 s. Gently remove excess while maintaining positive pressure (2 min). Final cure each surface for 20 s.
Predicta Bioactive Cement (shade A2)	Pr	Bioactive, self-adhesive dual-cure resin cement	Parkell, Edgewood, NY, USA (#23017)	Dual-paste syringe with auto-mix tips: Base component: glass oxide, Bis-GMA, UDMA, HEMA, TMPTMA, BTHQ, calcium fluoride, photoinitiators. Catalyst component: 10-MDP, HEMA, UDMA, TMPTMA, cumene hydroperoxide, photoinitiators	Use auto-mix tip, dispense into restoration, and tack light cure for 1–2 s. Gently remove excess. Maintain pressure and light cure for 20 s.

Here, Abbr.: Abbreviations; SiO_2:_ silicone oxide; Al_2_O_3_: aluminum oxide; Na_2_O: sodium oxide; K_2_O: potassium oxide; CaO: calcium oxide; TiO_2_: titanium oxide; Bis-GMA: bisphenol A diglycidyl methacrylate; BTHQ: 2,6-Di-tert-butyl-p-cresol; HEMA: hydroxyethyl methacrylate; MDP: 10-methacryloyloxydecyl dihydrogen phosphate; TEGDMA: triethyleneglycol dimethacrylate; UDMA: urethane dimethacrylate; TMPTMA: trimethylolpropane trimethacrylate.

The mold was placed over a glass slab, and the RCs were filled into the hollow, ensuring a controlled amount of material was used. A 1 mm thick glass slide was placed on top with gentle positive pressure; then the specimens were light-cured using a light-emitting diode (LED) curing unit (E-Morlit, Apoza, New Taipei, Taiwan), with an ensured irradiance of 1200 mW/cm^2^, from the top and bottom surfaces. The glass slide and Teflon mold were removed, and excess material at the specimen’s edges was cut with a No. 11 scalpel. Visual inspection of the specimens using 2.5× magnification and adequate lighting was performed to ensure the inclusion of only defect-free RC specimens. All RC specimens were kept in distilled water at room temperature, in a dark container, for 24 h to finish post-curing polymerization. Each disk-shaped specimen was measured for thickness with a digital micrometer (±0.01 mm) (Vernier caliper; Hi-Wendy, New Taipei, Taiwan) to ensure it was 1.0 ± 0.1 mm. The bottom was labeled, and the top was left clear for color measurements.

### 2.4. Staining Procedure

All specimens were placed in a coffee solution (Nescafé classic, Nestle, Vevey, Switzerland) after baseline color readings. The coffee solution was prepared by dissolving 2 g of coffee powder in 200 mL of boiled distilled water following the manufacturer’s recommendation. It was then allowed to reach room temperature before immersing the specimens and storing in an incubator at room temperature for 24 days total, and the solutions were refreshed daily to avoid mold and/or change in drink consistency. According to the coffee drink manufacturer and a study by Ertas et al. [39], the average coffee drinker has about 3.2 cups of coffee a day, spending around 15 min drinking each cup. So, storing coffee for 24 h is like drinking coffee for almost a month. This means that immersing for 24 days would correspond to 24 months of intraoral use.

### 2.5. Color Evaluation

For the VM/RC specimens, a plate measuring 10 × 10 × 5 mm was fabricated from a pre-sintered lithium silicate ceramic (GC initial LiSi blocks (shade A2), GC Corporation, Tokyo, Japan) to serve as the substrate and to imitate the underlying tooth structure. For the RC specimens, all color readings were completed against a white background.

The color reading was conducted by placing the tip of a VITA Easyshade Advance handheld spectrophotometer (VITA Zahnfabrik, Bad Säckingen, Germany) [3] at the center of the VM surface, not connected to the RC of each VM/RC specimen, and at that of the top surface of each RC specimen. The lighting conditions (illuminant D65) were kept constant for all color readings across groups. Color readings were completed following the Commission Internationale de l’Éclairage (CIE) CIELab* and CIEDE2000 color coordinate systems [40,41] at baseline and then after staining. ${\Delta E}_{00}$ was calculated using the following formula:

$${\Delta E}_{00}=\sqrt{{\left(\frac{\Delta L^{\prime }}{K_{L}S_{L}}\right)}^{2}+{\left(\frac{\Delta C^{\prime }}{K_{C}S_{C}}\right)}^{2}+{\left(\frac{\Delta H^{\prime }}{K_{H}S_{H}}\right)}^{2}+R_{T}\left(\frac{\Delta C^{\prime }}{K_{C}S_{C}}\right)\left(\frac{\Delta H^{\prime }}{K_{H}S_{H}}\right)}$$

where Δ*L*′, Δ*C*′, and Δ*H*′ denote changes in lightness, chroma, and hue, respectively; S_L_, S_C_, and S_H_ are the weighting functions for these coordinates; k_L_, k_C_, and k_H_ are the parametric weighting factors; and R_T_ is a rotation term accounting for hue and chroma interactions.

The CIEL*a*b* color coordinate differences (∆L, ∆a, and ∆b) were calculated. L* represents lightness (100 for white, 0 for black); a* indicates redness (positive) and greenness (negative); b* denotes yellowness (positive) and blueness (negative). The whiteness index formula was used to calculate the change in whiteness (∆WI_D_) [3,42]:

$${\Delta WI}_{D}=0.511{\Delta L}^{*}-2.324{\Delta a}^{*}-1.100{\Delta b}^{*}$$

### 2.6. Statistical Analysis

A priori power analysis was conducted using G*Power software (G*Power Version 3.1.9.7, Franz Faul, Universität Kiel, Germany) for a two-way and one-way analysis of variance (ANOVA), indicating a total sample size of 90 specimens across 9 groups (f ~0.6 and power~90%). Statistical analysis was performed using DATAtab (DATAtab e.U., Graz, Austria [43]) and R software (Version 4.5.0 for Mac, R Foundation for Statistical Computing, Vienna, Austria [44]). Normality and homogeneity were confirmed using the Kolmogorov–Smirnov and Levene tests (*p* = 0.42, 0.066, respectively). A two-way ANOVA with Bonferroni post hoc comparisons was used to assess the effects of resin cement material (RC material) and ceramic thickness on ΔE_00_ and ΔWID, as well as their interaction. A one-way ANOVA was applied to analyze color change in the RC groups (α < 0.05).

## 3. Results

### 3.1. Results for VM/RC Groups

The two-way ANOVA showed that there was a significant difference between the groups of the independent variable RC material in relation to the dependent variables ΔE_00_ and ΔWI_D_ (*p* < 0.001, η2p = 0.92, 0.63, respectively); that there was no significant difference between the groups of the independent variable ceramic thickness after adjusting for multiple comparisons in relation to the dependent variables ΔE_00_ and ΔWI_D_ (*p* = 0.51, 0.23, respectively); and that there was a significant interaction between the two variables RC material and ceramic thickness in relation to the dependent variables ΔE_00_ and ΔWI_D_ (*p* < 0.001, 0.61, 0.49, respectively). This indicated that the effect of ceramic thickness on ΔE_00_ and ΔWI_D_ is RC material-dependent. Ac VM/RC specimens had a significantly greater ΔE_00_ than both Pn and Pr (*p* < 0.001), and Pr had a slightly significantly higher ΔE_00_ than Pn (*p* = 0.04). On the other hand, Pn had a significantly lower change in whiteness (ΔWI_D_) than Pr and Ac (*p* < 0.001), but there was no significant difference between Pn and Pr (*p* = 0.8).

**Figure 3:**
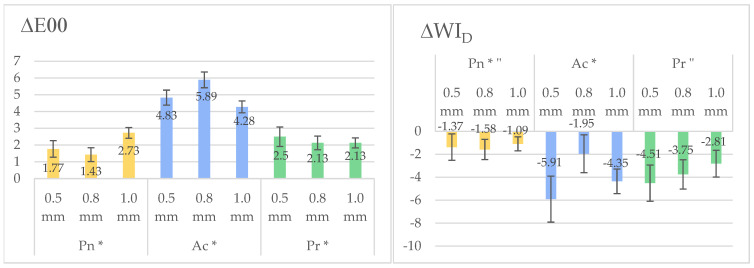
A graphical representation showing the mean color change (ΔE_00_) and change in whiteness index (ΔWI_D_) for the VM/RC groups at the three different ceramic thicknesses. The black error bars indicate standard deviations. The same superscript symbols (* and ″) designate significantly different mean values in the Bonferroni test for the independent variable RC material (*p* < 0.05).

The mean ΔE_00_ and ΔWI_D_ values for the different VM/RC groups with the Bonferroni test results for significant differences for the independent variable RC material are shown in Figure 3 and Table A1.

Ac consistently showed the highest ΔE_00_ across all ceramic thickness levels. Since the interaction between RC material and ceramic thickness was significant, the color and whiteness changes were compared across different ceramic thicknesses within each RC material. Pn specimens at a ceramic thickness of 1.0 mm showed a statistically significantly greater ΔE_00_ than the other two ceramic thicknesses (*p* < 0.05) but a greater ΔWI_D_ at 0.5 mm than at 1.0 mm. Ac specimens showed a significantly greater ΔE_00_ at a ceramic thickness of 0.8 mm compared to 0.5 and 1.0 mm (*p* < 0.001), but they showed a greater ΔWI_D_ at 1.0 mm than at 0.8 mm. Pr showed no significant effect of ceramic thickness on ΔE_00_ and ΔWI_D_ (*p* > 0.05).

When comparing the different RCs at the same ceramic thickness, Pn specimens at a ceramic thickness of 0.5 and 0.8 mm showed significantly lower ΔE_00_ and ΔWI_D_ than Pr and Ac (*p* < 0.001), while Ac had a greater ΔE_00_ at a ceramic thickness 1.0 mm than both Pr and Pn (*p* < 0.01). At 0.8 mm ceramic thickness, Pr specimens showed a greater ΔWI_D_ than Pn (*p* < 0.001). The detailed mean, standard deviation, minimum, maximum, and confidence interval) for ΔE_00_, ΔWI_D_, ΔL*, Δa*, and Δb* with the statistically significant results for the pairwise comparison between the different ceramic thickness groups within each RC material are shown in Table 2.

### 3.2. Results for RC Groups

After the confirmation of the normal distribution of the data, a one-way ANOVA was conducted for the variables ΔE_00_, ΔWI_D_, ΔL*, Δa*, and Δb* at *p* < 0.05. The one-way ANOVA revealed a significant difference in ΔE_00_, ΔWI_D_, ΔL*, and Δa* between RCs (*p* < 0.01, η2p = 0.48 (ΔE_00_), 0.63 (ΔWI_D_)). However, there was no statistically significant difference in Δb* between the three investigated RCs (*p* = 0.15).

The Bonferroni post hoc test revealed that the ΔE_00_ of Pr was significantly lower than that of Pn (*p* < 0.001) and Ac (*p* = 0.003). The mean ΔWI_D_ of Ac was significantly greater than that of both Pn (*p* = 0.03) and Pr (*p* = 0.005). The mean ΔL* of Pr was significantly lower than that of both Pn (*p* = 0.001) and Ac (*p* = 0.02). The mean Δa* of Ac was significantly lower than that of Pn and Pr (*p* < 0.001), and the mean Δa* value for Pn was significantly higher than that for Pr (*p* = 0.02). The detailed mean and standard deviation values for the investigated variables in the three investigated RCs are shown in Table 3.

## 4. Discussion

The present study investigated the effects of bioactive resin cements (RCs) on the color stability and final shade of a CAD/CAM glass–ceramic material at three different ceramic thicknesses. The study results demonstrated significant differences in color stability among the tested RCs, between the ceramic specimens bonded to the different RCs, and between the different thickness levels depending on the RC used. Therefore, the null hypothesis that there is no significant difference in the color stability between the two bioactive (Pr and Ac) and the Pn non-bioactive cements had to be rejected. Additionally, the hypotheses that there is no significant effect of ceramic thickness on the final color and whiteness of the bonded VM glass–ceramic and that the type of RC does not significantly affect the final color and whiteness of the bonded VM material were rejected.

In the current study, the 1 mm thick resin cement is much greater than what is recommended in the literature for the cementation of crowns (50–100 μm) [26,45] to isolate the cement’s contribution without interference from the underlying tooth structure. This was selected based on a previously published study protocol [19] and to ensure enough resin thickness to magnify optical differences encountered during the study and reach reliable results, as per ISO and spectrophotometric testing standards [46]. Clinically relevant cement films of 100 µm or less would have likely produced smaller ∆E_00_ and ∆WI_D_ changes [19,26]; however, the overall trends observed in the current study would likely persist. Additionally, the color readings for the VM/RC groups were taken against a lithium silicate ceramic in shade A2, which is reportedly the most commonly used shade in dentistry [47]. The same ceramic plate was used for all specimens to maintain standardization in readings between all samples and avoid the effects of staining or aging on the substrate interfering with the actual readings of the specimen materials. The same ceramic plate was used for all specimens to ensure consistency. Color readings were taken from the central area of each sample, ensuring consistent readings of an isolated part without edge loss effects.

The interaction between resin cement properties and CAD/CAM glass–ceramic restorations represents a critical factor in achieving optimal esthetic outcomes in modern dentistry. The translucency of CAD/CAM glass–ceramics makes them especially vulnerable to color effects from what is underneath substrates [48]. In the current study, all specimens showed a color change, which could be caused by coffee stain adsorption, as documented in previous studies [32,36,49,50]. The bioactive cements demonstrated varying degrees of impact on the final ceramic appearance. Ac showed the most pronounced effect, with significant, visually detectable color changes that are seen through the ceramic layer, especially with a bonded 0.8 mm thick glass–ceramic layer. This could be attributed to its high water sorption and its unique rubberized matrix combined with bioactive fillers. These components increase water permeability and plasticization, while the bioactive fillers may alter the polymer network and further promote discoloration over time [51]. The whiteness index (∆WI_D_) varied significantly among RCs, with Ac-bonded ceramic specimens showing the greatest change in the whiteness index (−5.91 ± 2 with a ceramic thickness of 0.5 mm) and Pr demonstrating better ΔWI_D_ stability (−2.81 ± 1.17 with a ceramic thickness of 1.0 mm). This further showed the greater deterioration in Ac’s color, suggesting that certain bioactive formulations may compromise the color stability of thin, highly translucent restorations commonly used in laminate veneers. These findings also align with previous studies, which have shown the greater discoloration susceptibility of Ac [32,51,52,53].

While ceramic thickness (0.5–1.0 mm) did not significantly affect ∆E_00_ or ∆WI_D_ overall (*p* = 0.51, 0.23), important interactions between ceramic thickness and RC material were detected, suggesting that the ceramic thickness effect is RC material type-dependent. The material type dependency of the color change in cemented ceramic veneers was also documented in a recent study by Miletic et al. [54]. Additionally, a previous study found that the shade of glass–ceramics is highly dependent on its thickness [55]. In the present study, Ac’s mean ∆E_00_ reached a maximum at a ceramic thickness of 0.8 mm (5.89 ± 0.47), which could indicate its filler’s optical properties interacting with the intermediate ceramic translucency of the bonded VM. Ac may have a higher opacity than the other RCs, which could partially mask the substrate, scatter light differently, and reduce ceramic contribution and may, therefore, pose a higher risk of mismatching the restoration’s intended shade [27]. In contrast to 1.0 mm ceramic thickness, where VM’s own opacity may have overshadowed Ac’s contribution, the 0.8 mm thickness would have allowed more light to interact with Ac, amplifying the resultant ∆E_00_. On the other hand, Pn showed a higher ∆E_00_ at a ceramic thickness of 1.0 mm (2.73 ± 0.32) compared to 0.5 mm (1.77 ± 0.49) (*p* < 0.05), likely due to increased light scattering in thicker ceramics amplifying underlying cement discoloration [26,55]. This demonstrated that even at 1.0 mm thickness, certain cement formulations, particularly Ac, still caused noticeable color change, highlighting that thickness alone cannot fully offset cement-related effects. The greater change in deterioration in the whiteness index would potentially make teeth appear less vibrant over time.

The color changes seen in the current study for resin cement and CAD/CAM glass–ceramic with the resin cement background must be evaluated through the lens of established perceptibility and acceptability thresholds in dental color science, specifically 0.8 for perceptibility (PT) and 1.8 for acceptability (AT) [56,57]. The color change in all RC and VM/RC specimens was above both AT and PT, except for Pn’s 0.8 mm ceramic thickness group, where the ΔE_00_ value was slightly below AT but still above PT. This indicated that the color change produced by the resin cements would be both visibly apparent and clinically unacceptable to trained observers [58]. In the current study, Ac bioactive cement produced ΔE_00_ values ranging from 4.28 to 5.89 when bonded to different glass–ceramic thicknesses and 4.83 when the resin was completely exposed to the staining solution. These values were 3–6 times higher than clinical AT, representing drastic color changes that would be noticeable not only to clinicians but also to patients. This study also revealed that color changes were not uniform across color parameters. Ac cement exhibited especially large shifts in the red–green (a*) color parameter, reaching up to 3.62, which human vision is particularly sensitive to detecting [52]. This explains why, even at a thickness of 1.0 mm, the color changes remained clinically unacceptable, despite the thicker ceramic layer.

From a biomimetic perspective, the current study’s results suggest that while bioactive cements offer biological benefits [3,8], their optical properties may compromise the natural tooth-mimicking appearance of CAD/CAM glass–ceramics, such as the one tested in the current study (VITA Mark II). Clinicians should carefully weigh bioactive benefits against esthetic drawbacks when choosing these cements for anterior esthetic restorations. Furthermore, the significant RC/VM thickness interaction encountered in this study highlighted the especially critical importance of cement selection for thin ceramics, where opaque RCs risk greater color shifts in the final restoration. The results of the current study might be helpful in predicting the performance of other glass–ceramic systems with similar translucency characteristics; however, a further investigation of RC color effect on other ceramic materials compared to VM is recommended. Future research should explore the interactions in other ceramic materials and under longer aging conditions, different staining media, thinner cement film thicknesses, and different cement shades, to predict the long-term esthetic performance of bonded restorations with and without bioactive properties. The current findings align with previous reports on the susceptibility of bioactive cements to discoloration, but they contrast with those highlighting their improved color stability. The differences may stem from variations in experimental protocols or material formulations. While this study provides valuable insights, its limitations include the in vitro setting without thermal cycling, the use of coffee as the sole staining agent, the use of a thick cement film, and the absence of salivary effects such as viscosity, flow, and pH, which may limit its generalizability to clinical conditions. The current study’s evaluation of only one glass–ceramic material and one cement shade may also limit the generalizability of the study results to other cement shades and ceramics with variable translucencies, such as zirconia and lithium disilicates. Future studies to validate the observed color change trends obtained in this study at clinically relevant cement thicknesses are recommended to enhance translational relevance. Additional future studies may benefit from integrating broader perspectives, such as mechanical and interfacial behavior, to further understand the interaction between resin-based cements and advanced ceramic materials [59]. Furthermore, long-term clinical trials are recommended to validate these findings in real-world scenarios.

## 5. Conclusions

Within the limitations of this in vitro study, the findings suggest that the type of resin cement (RC) plays a crucial role in the color and whiteness stability of CAD/CAM VITA Mark II (VM) glass–ceramic restorations following staining. Bioactive RCs exhibited greater color change compared to the conventional RC, with Activa cement (Ac) showing the highest level of discoloration. Although ceramic thickness alone did not have a significant influence, its interaction with RC type affected the overall results, particularly under thinner restorations.

## Figures and Tables

**Table 2:** Descriptive statistics for change in color (ΔE_00_), whiteness index (ΔWI_D_), and the CIE Lab color coordinates (ΔL, Δa*, and Δb*) of the VM/RC groups. Values with the same lowercase superscripts are statistically significantly different ΔE_00_ and ΔWI_D_ within each RC material at different thicknesses (*p* < 0.05). Values with the same uppercase superscripts are statistically significantly different ΔE_00_ and ΔWI_D_ within each ceramic thickness with different RC materials (*p* < 0.05). Statistical differences were calculated only for ΔE_00_ and ΔWI_D_.

	RC	VM Thickness	n	Mean ± Std.	Min.	Max.	95% Confidence Interval
ΔE_00_	Pn	0.5 mm	10	1.77 ± 0.49 ^aAB^	0.99	2.34	1.42–2.12
		0.8 mm	10	1.43 ± 0.41 ^bCD^	0.61	1.9	1.14–1.73
		1.0 mm	10	2.73 ± 0.32 ^abEF^	2.17	3.31	2.49–2.96
	Ac	0.5 mm	10	4.83 ± 0.45 ^cA^	4.21	5.39	4.5–5.15
		0.8 mm	10	5.89 ± 0.47 ^cdC^	5.06	6.61	5.55–6.23
		1.0 mm	10	4.28 ± 0.35 ^dE^	3.78	4.91	4.03–4.53
	Pr	0.5 mm	10	2.5 ± 0.58 ^AB^	1.57	3.46	2.09–2.92
		0.8 mm	10	2.13 ± 0.41 ^CD^	1.6	2.78	1.84–2.42
		1.0 mm	10	2.13 ± 0.3 ^EF^	1.72	2.72	1.91–2.34
ΔWI_D_	Pn	0.5 mm	10	−1.37 ± 1.15 ^efG^	−0.72	3.19	0.54–2.19
		0.8 mm	10	−1.58 ± 0.88 ^eE^	−2.81	0.13	−2.21–−0.95
		1.0 mm	10	−1.09 ± 0.61 ^fF^	−2.13	−0.39	−1.52–−0.66
	Ac	0.5 mm	10	−5.91 ± 2 ^gG^	−10.6	−3.89	−7.34–−4.48
		0.8 mm	10	−1.95 ± 1.65 ^gh^	−4.62	0.48	−3.13–−0.78
		1.0 mm	10	−4.35 ± 1.07 ^hF^	−6.03	−2.24	−5.11–−3.58
	Pr	0.5 mm	10	−4.51 ± 1.58 ^G^	−6.76	−2.46	−5.64–−3.38
		0.8 mm	10	−3.75 ± 1.28 ^E^	−5.81	−2.1	−4.66–−2.83
		1.0 mm	10	−2.81 ± 1.17	−4.3	−1.05	−3.65–−1.97
ΔL*	Pn	0.5 mm	10	2.32 ± 0.77	0.9	3.2	1.77–2.87
		0.8 mm	10	1.23 ± 0.54	0.4	1.7	0.84–1.62
		1.0 mm	10	3.4 ± 0.39	2.8	4.1	3.12–3.68
	Ac	0.5 mm	10	0.41 ± 0.26	0.1	0.9	0.22–0.6
		0.8 mm	10	2.35 ± 0.71	1.2	3.5	1.84–2.86
		1.0 mm	10	0.54 ± 0.67	0	2.3	0.06–1.02
	Pr	0.5 mm	10	0.95 ± 0.88	0	3	0.32–1.58
		0.8 mm	10	0.38 ± 0.32	0.1	1.1	0.15–0.61
		1.0 mm	10	0.9 ± 0.69	0	2.3	0.4–1.4
Δa*	Pn	0.5 mm	10	0.21 ± 0.24	−0.1	0.6	0.04–0.38
		0.8 mm	10	0.76 ± 0.28	0.2	1.1	0.56–0.96
		1.0 mm	10	0.99 ± 0.16	0.8	1.3	0.88–1.1
	Ac	0.5 mm	10	3.49 ± 0.31	3	4	3.27–3.71
		0.8 mm	10	3.62 ± 0.41	2.7	4.2	3.33–3.91
		1.0 mm	10	2.97 ± 0.27	2.6	3.3	2.78–3.16
	Pr	0.5 mm	10	1.61 ± 0.41	0.7	2.1	1.31–1.91
		0.8 mm	10	1.53 ± 0.27	1.2	2	1.34–1.72
		1.0 mm	10	1.45 ± 0.25	1.1	1.8	1.27–1.63
Δb*	Pn	0.5 mm	10	−0.61 ± 0.71	−1.4	0.5	−1.12–−0.1
		0.8 mm	10	0.4 ± 0.58	−0.4	1.4	−0.02–0.82
		1.0 mm	10	0.48 ± 0.52	−0.4	1.1	0.11–0.85
	Ac	0.5 mm	10	−1.81 ± 1.62	−3.6	1.6	−2.97–−0.65
		0.8 mm	10	−4.78 ± 0.88	−6.6	−3.6	−5.41–−4.15
		1.0 mm	10	−2.07 ± 0.44	−2.9	−1.4	−2.39–−1.75
	Pr	0.5 mm	10	1.14 ± 0.72	−0.4	1.8	0.63–1.65
		0.8 mm	10	0.35 ± 0.78	−0.7	1.9	−0.21–0.91
		1.0 mm	10	−0.09 ± 0.63	−1.3	0.8	−0.54–0.36

Here, RC is resin cement, ΔE_00_ is color change, ΔWI_D_ is whiteness index change, ΔL* is change in lightness, Δa* is change in red–green color dimension, Δb* is change in yellow–blue color dimension, and std is standard deviation.

**Table 3:** Descriptive statistics for change in color (ΔE_00_), whiteness index (ΔWI_D_), and the CIE Lab color coordinates (ΔL, Δa*, and Δb*) of the resin cement (RC) groups. Values with the same uppercase superscripts are statistically significantly different (*p* < 0.05).

	RC	n	Mean ± Std.	Min.	Max.	95% Confidence Interval for Mean
ΔE_00_	Pn	10	5.21 ± 0.91 ^A^	4.08	6.66	4.56–5.86
	Ac	10	4.79 ± 0.98 ^B^	3.36	5.95	4.08–5.49
	Pr	10	3.33 ± 0.77 ^AB^	2.46	4.79	2.78–3.87
ΔWI_D_	Pn	10	−1.82 ± 3.66 ^C^	−10.89	1.4	−4.44–0.8
	Ac	10	2.12 ± 3.25 ^CD^	−5.04	6.12	−0.21–4.44
	Pr	10	−2.86 ± 2.5 ^D^	−6.09	3.27	−4.65–−1.08
ΔL*	Pn	10	6.99 ± 1.57 ^E^	3.7	9	5.87–8.11
	Ac	10	6.22 ± 1.63 ^F^	3.8	8.1	5.05–7.39
	Pr	10	4.1 ± 1.49 ^EF^	2.2	6.9	3.04–5.16
Δa*	Pn	10	1.81 ± 0.64 ^G^	1.2	3.1	1.35–2.27
	Ac	10	−0.66 ± 0.51 ^G^	−1.3	0.4	−1.03–−0.29
	Pr	10	1.14 ± 0.33 ^G^	0.3	1.4	0.91–1.37
Δb*	Pn	10	1.08 ± 1.88	−0.7	5.7	−0.27–2.43
	Ac	10	2.36 ± 1.44	0.1	5.5	1.33–3.39
	Pr	10	2.1 ± 1.03	−0.4	3.6	1.36–2.84

Here, RC is resin cement, ΔE_00_ is color change, ΔWI_D_ is whiteness index change, ΔL* is change in lightness, Δa* is change in red–green color dimension, Δb* is change in yellow–blue color dimension, and std is standard deviation.

## Data Availability

The raw data supporting the conclusions of this article will be made available by the authors on request.

## Abbreviations

The following abbreviations are used in this manuscript:

Ac	ACTIVA BioACTIVE cement
Al_2_O_3_	Aluminum oxide
ANOVA	One-way analysis of variance
AT	Acceptability threshold
Bis-GMA	Bisphenol A diglycidyl methacrylate
BTHQ	2,6-di-tert-butyl-p-cresol
CaO	Calcium oxide
CIE	Commission International de L’Eclairage
HEMA	Hydroxyethyl methacrylate
ISO	International Organization for Standardization
K_2_O	Potassium oxide
LED	Light-emitting diode
MDP	Methacryloyloxydecyl dihydrogen phosphate
Na_2_O	Sodium oxide
Pn	Panavia resin cement
Pr	Predicta Bioactive Cement
PT	Perceptibility threshold
RC	Resin cement
SiO_2_	Silicone oxide
TEGDMA	Triethyleneglycol dimethacrylate
TiO_2_	Titanium oxide
TMPTMA	Trimethylolpropane trimethacrylate
UDMA	Urethane dimethacrylate
VM	Vita Mark II

## Appendix A

**Table A1:** The analysis of variance (ANOVA) and Bonferroni pairwise comparisons of the change in color (ΔE_00_) and whiteness (ΔWI_D_) as the main study outcomes, including effect sizes and 95% confidence intervals.

VM/RC Specimens								
Two-Way ANOVA ΔE_00_								
		Type III Sum of Squares	df	Mean Square	F	* p *	η 2p	95% CI
RC		167.49	2	83.75	457.33	<0.001	0.92	0.87- 0.95
VM thickness		0.25	2	0.12	0.68	0.511	0.02	0.00- 0.10
RC × VM thickness		23.08	4	5.77	31.52	<0.001	0.61	0.45- 0.72
Error		14.83	81	0.18				
** The Bonferroni post hoc test **								
** VM thickness—RC **			** Mean Difference **	** SE **	** t **	*** p ***	** 95% CI **	** Cohen’s d **
0.5 mm—Pn		0.8 mm—Pn	0.34	0.191	1.76	1	−0.22, 0.90	0.80
0.5 mm—Pn		1.0 mm—Pn	−0.95	0.191	−4.99	<0.001	−1.51, −0.39	−2.24
0.8 mm—Pn		1.0 mm—Pn	−1.29	0.191	−6.75	<0.001	−1.85, −0.73	−3.04
0.5 mm—Pr		0.8 mm—Pr	0.38	0.191	1.97	1	−0.18, 0.94	0.90
0.5 mm—Pr		1.0 mm—Pr	0.38	0.191	1.96	1	−0.18, 0.94	0.90
0.8 mm—Pr		1.0 mm—Pr	0	0.191	−0.01	1	−0.56, 0.56	0.00
0.5 mm—Ac		0.8 mm—Ac	−1.06	0.191	−5.55	<0.001	−1.62, −0.50	−2.50
0.5 mm—Ac		1.0 mm—Ac	0.55	0.191	2.86	0.195	−0.01, 1.11	1.30
0.8 mm—Ac		1.0 mm—Ac	1.61	0.191	8.41	<0.001	1.05, 2.17	3.80
**Two-Way ANOVA ΔWI_D_**								
		** Type III Sum of Squares **	** df **	** Mean Square **	** F **	*** p ***	** η2p **	**95% CI**
RC		239.78	2	119.89	68.09	<0.001	0.63	0.50- 0.72
VM thickness		5.26	2	2.63	1.49	0.231	0.04	0.00- 0.12
RC × VM thickness		138.54	4	34.63	19.67	<0.001	0.49	0.35- 0.60
Error		142.63	81	1.76				
** The Bonferroni post hoc test **								
** VM thickness—RC **			** Mean Difference **	** SE **	** t **	*** p ***	** 95% CI **	** Cohen’s d **
0.5 mm—Pn		0.8 mm—Pn	2.95	0.593	4.96	<0.001	1.22, 4.68	2.89.89
0.5 mm—Pn		1.0 mm—Pn	2.46	0.593	4.15	0.003	0.73, 4.19	2.67
0.8 mm—Pn		1.0 mm—Pn	−0.49	0.593	−0.82	1	−2.22, 1.24	−0.64
0.5 mm—Pr		0.8 mm—Pr	−0.76	0.593	−1.29	1	−2.49, 0.97	−0.53
0.5 mm—Pr		1.0 mm—Pr	−1.7	0.593	−2.86	0.192	−3.43, 0.03	−1.22
0.8 mm—Pr		1.0 mm—Pr	−0.94	0.593	−1.58	1	−2.67, 0.79	−0.76
0.5 mm—Ac		0.8 mm—Ac	−3.96	0.593	−6.67	<0.001	−5.69, −2.23	−2.16
0.5 mm—Ac		1.0 mm—Ac	−1.56	0.593	−2.63	0.367	−3.29, 0.17	−0.97
0.8 mm—Ac		1.0 mm—Ac	2.4	0.593	4.04	0.004	0.67, 4.13	1.73
**RC specimens**								
**One-Way ANOVA ΔE** **_00_**								
		** Type III Sum of Squares **	** df **	** Mean Square **	** F **	*** p ***	** η2p **	** 95% CI **
RC		19.59	2	9.79	12.4	<0.001	0.48	0.21- 0.64
Residual		21.32	27	0.79				
Total		40.91	29					
** The Bonferroni post hoc test **								
	** Mean diff. **		** Std. Error **	** t **	*** p ***	** 95% CI lower limit **	** 95% CI upper limit **	** Cohen’s d **
Pn—Pr	1.89		0.397	4.75	<0.001	0.86	2.91	2.12
Pn—Ac	0.43		0.397	1.07	0.88	−0.6	1.45	0.48
Pr—Ac	−1.46		0.397	−3.68	0.003	−2.48	−0.44	−1.64
**One-Way ANOVA ΔWI_D_**								
		** Type III Sum of Squares **	** df **	** Mean Square **	** F **	*** p ***	** η2p **	** 95% CI **
RC		138.02	2	69.01	6.85	0.004	0.34	0.08- 0.52
Residual		272.18	27	10.08				
Total		410.2	29					
** The Bonferroni post hoc test **								
	** Mean diff. **		** Std. Error **	** t **	** p **	** 95% CI lower limit **	** 95% CI upper limit **	** Cohen’s d **
Pn—Pr	1.04		1.42	0.73	1	−2.61	4.69	0.33
Pn—Ac	−3.94		1.42	−2.77	0.03	−7.59	−0.29	−1.24
Pr—Ac	−4.98		1.42	−3.51	0.005	−8.63	−1.33	−1.57
